# Timeliness and completeness of measles vaccination among children in rural areas of Guangxi, China: A stratified three-stage cluster survey

**DOI:** 10.1016/j.je.2016.08.006

**Published:** 2017-02-08

**Authors:** Xianyan Tang, Alan Geater, Edward McNeil, Hongxia Zhou, Qiuyun Deng, Aihu Dong

**Affiliations:** a Department of Epidemiology & Biostatistics, School of Public Health, Guangxi Medical University, Guangxi Zhuang Autonomous Region, China; b Epidemiology Unit, Faculty of Medicine, Prince of Songkla University, Hat Yai, Thailand; c Institute of Vaccination, Guangxi Center for Disease Control and Prevention, Guangxi Zhuang Autonomous Region, China

**Keywords:** Measles, Vaccination, Timeliness, Completeness, Rural children

## Abstract

**Background:**

Large-scale outbreaks of measles occurred in 2013 and 2014 in rural Guangxi, a region in Southwest China with high coverage for measles-containing vaccine (MCV). This study aimed to estimate the timely vaccination coverage, the timely-and-complete vaccination coverage, and the median delay period for MCV among children aged 18–54 months in rural Guangxi.

**Methods:**

Based on quartiles of measles incidence during 2011–2013, a stratified three-stage cluster survey was conducted from June through August 2015. Using weighted estimation and finite population correction, vaccination coverage and 95% confidence intervals (CIs) were calculated. Weighted Kaplan–Meier analyses were used to estimate the median delay periods for the first (MCV1) and second (MCV2) doses of the vaccine.

**Results:**

A total of 1216 children were surveyed. The timely vaccination coverage rate was 58.4% (95% CI, 54.9%–62.0%) for MCV1, and 76.9% (95% CI, 73.6%–80.0%) for MCV2. The timely-and-complete vaccination coverage rate was 47.4% (95% CI, 44.0%–51.0%). The median delay period was 32 (95% CI, 27–38) days for MCV1, and 159 (95% CI, 118–195) days for MCV2.

**Conclusions:**

The timeliness and completeness of measles vaccination was low, and the median delay period was long among children in rural Guangxi. Incorporating the timeliness and completeness into official routine vaccination coverage statistics may help appraise the coverage of vaccination in China.

## Highlights

• The timeliness and completeness of measles vaccination was low.
• The median delay period of measles vaccination was long among Guangxi children.
• Regions with higher measles incidence had higher rates of untimely coverage.

## Introduction

Measles is a highly contagious disease and one of the major causes of death among children worldwide.^1^ Vaccination with measles-containing vaccine (MCV) is the most effective way to reduce the morbidity and mortality associated with the disease. Due to worldwide measles vaccination since the 1980s, the reported coverage of measles vaccination across most regions has reached 90% and is as high as 95% in developed countries.^2^ However, large-scale outbreaks of measles still occur. In 2010, a total of 33 countries in Europe reported measles outbreaks.^3^, ^4^, ^5^, ^6^, ^7^ Most measles cases were aged <12 months or 15–29 years and were either unvaccinated or vaccinated incompletely.^4^, ^6^, ^8^ Thus, the goal of measles elimination was not met in WHO Europe Region (WHO/EUR) by 2010.^9^ Similarly, outbreaks of measles frequently occur in WHO Western Pacific Region (WHO/WPR), a region with high measles vaccination coverage.^10^, ^11^, ^12^, ^13^

China is one of the member nations in WHO/WPR and accounts for 75% of the region's population. Since 2003, approximately 70% of the reported measles cases in WHO/WPR were from China.^14^ Thus, the progress towards measles elimination in China is critical for achieving the 2012 measles elimination goal in WHO/WPR.^2^, ^15^ Although the reported measles vaccination coverage rate in China has been over 90% since 2006, measles outbreaks still occurred in several provinces, including Guangxi, Beijing, Zhejiang, and Shangdong Provinces.^16^, ^17^, ^18^, ^19^

Since 2007, Guangxi has reached 95% coverage for both the first (MCV1) and second (MCV2) dose of measles vaccine, but large-scale measles outbreaks still occurred between 2013 and 2014.^20^, ^21^, ^22^ Of the 1341 and 3167 notifiable measles cases in 2013 and 2014, 71% and 56%, respectively, were scattered children in rural areas, and 60% and 50%, respectively, were children aged less than 24 months (Data from the Notifiable Infectious Disease Surveillance System in Guangxi Center for Disease Control [CDC]). Among all measles cases, the vaccination coverage rates in 2013 and 2014 were 63% and 51%, respectively; among cases aged 8–12 months, the respective timely vaccination coverage rates for MCV1 were only 23% and 15%. Moreover, high-incidence regions were clustered in West and Southwest Guangxi (i.e., Baise, Chongzuo, Hechi, and Nanning Prefectures). According to WHO recommendation, completeness of measles vaccination means that children should receive the two doses of MCV (i.e., MCV1 and MCV2) so as to prevent outbreaks of measles.^23^ Thus, the measles outbreaks may reflect a lack of herd immunity against measles among children in rural Guangxi, which implies that complete measles vaccination might not be routinely delivered to susceptible populations on time.

In theory, high reported measles vaccination coverage (>95%) should protect children against measles.^24^ It is worth noting that the timeliness of vaccination is not usually reported in the official vaccine coverage statistics.^25^ Reported coverage may mask substantial delays in vaccination and neglect the timeliness of vaccination.^26^ Therefore, timely vaccination may be a key to achieving elimination of measles. Here, we hypothesized that the timeliness of measles vaccination may be poor in measles-endemic areas of Guangxi.

Previous studies have revealed that untimely vaccination of measles poses a threat to susceptible children.^27^, ^28^, ^29^, ^30^, ^31^, ^32^, ^33^, ^34^, ^35^ However, most studies focused on urban children, and rural children were neglected. Few studies have assessed the timely vaccination for MCV2 or the median delay period, and no study has explored the timely-and-complete vaccination for MCV1 and MCV2. Furthermore, few studies have used complex sampling designs and weighted analysis methods to determine the measles vaccination coverage at the national or provincial level. Additionally, there is a paucity of studies on the timeliness and completeness of measles vaccination in China, particularly in western rural areas.

Therefore, a stratified three-stage cluster survey was conducted among children aged 18–54 months in rural Guangxi, with the aim of determining the timely vaccination coverage rate of MCV, the timely-and-complete vaccination coverage rate of MCV, and the median delay period of MCV using weighted estimators appropriate to the complex survey design. Results of this study may help provide a better understanding of the possible explanations for the large-scale outbreaks of measles in rural Guangxi, a region with high measles vaccination coverage.

## Methods

### Study design and setting

**Fig. 1:**
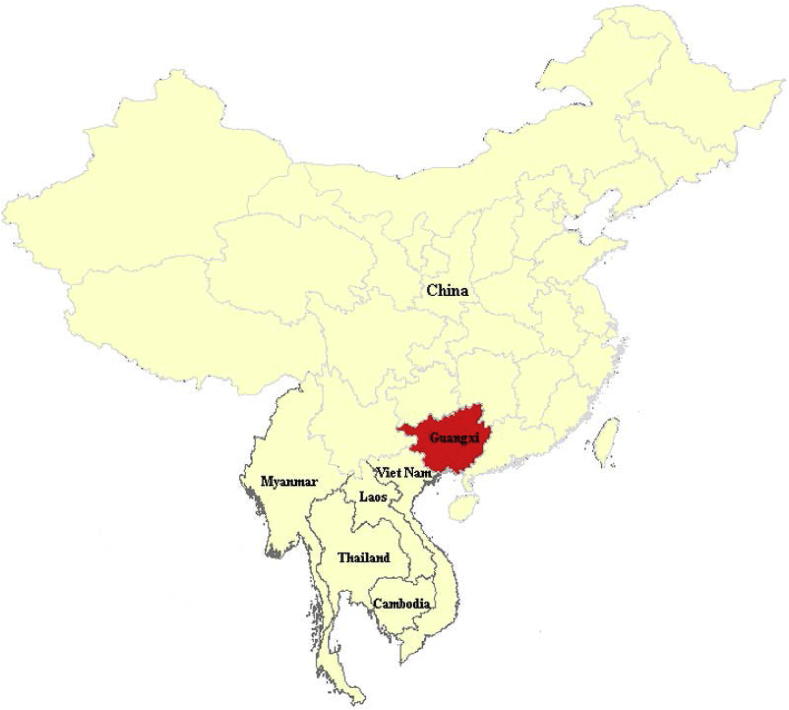
The location of Guangxi, China.

The present study was a cross-sectional multi-stage cluster survey, and the study setting was rural areas of Guangxi Zhuang Autonomous Region. Zhuang, the largest minority ethnicity of China, are mainly distributed in Guangxi, a typically mountainous area in Southwest China (Fig. 1). Guangxi is one of the five autonomous regions in China, where Zhuang, Han, Yao, and Miao ethnicities are the major residents. Guangxi consists of 14 prefectures and 109 counties, with an area of 236,700 km^2^ and a population of 46.8 million residents in 2012. Geographically, Guangxi has proximity to the Association of Southeast Asian Nations (ASEAN) member states. In this study, rural areas were defined as any county or county-level city. A total of 75 counties or county-level cities were classified into rural Guangxi.

### Study population

According to the national Expanded Programme on Immunization (EPI) in China, routine measles vaccination comprises two doses of MCV. Children should be vaccinated with MCV1 at the age of 8 months, and MCV2 should be given to children between the ages of 18 and 24 months.^26^ To determine the timeliness of measles vaccination among a child cohort having approximately the same age distribution as the measles cases, the population of interest was defined as children aged 18–54 months in rural Guangxi. Ideally, children in this age range should have received at least one dose of MCV.

The inclusion criteria for target children were: (1) living in rural Guangxi for at least 3 months, (2) age 18–54 months at the time of interview, (3) availability of child's vaccination certificate, and (4) the primary guardian of the child able to communicate verbally in local language or Mandarin Chinese without any barriers. We recruited investigators who could speak the local language fluently, and employed local vaccination practitioners to interview the primary guardian. Children were excluded if they had any contraindications for vaccination or if they received any dose of MCV outside of their hometown.

### Sample size

A total of at least 1200 children were required to estimate the timeliness of MCV with a precision of 4% and type I error rate of 5%. Given the reduction of sample efficiency and estimation precision under the cluster sampling design, a design effect of 2.0 was used.^36^ No previous information on the coverage of timely-and-complete MCV could be found, so we used an estimated 50% coverage.

WHO recommends that it is feasible to sample 30 clusters (villages) from each county.^37^ Thus, four counties with a total of 120 clusters were selected. In each county, 30 clusters and 10 children per cluster were sampled.

### Sampling technique and sampling procedure

The present study was a stratified three-stage cluster survey. In stratified sampling, all 75 rural counties were classified into four strata based on quartiles of measles incidence during 2011–2013. There were 17 counties in the first stratum, 12 counties in the second stratum, 21 counties in the third stratum, and 25 counties in the fourth stratum. From the first (lowest incidence) to fourth stratum (highest incidence), one county per stratum was randomly selected: Longan, Zhaoping, Wuxuan, and Longlin Counties. At the first stage of cluster sampling, five towns per county were randomly selected. At the second stage, six villages per town were randomly selected. At the third stage, ten children per village were randomly selected from a list obtained from the local township hospitals, which contains the number and names of all the children in the target age range in the area. If two or more eligible children were in the same household, only the youngest child was selected.

### Data collection and data management

The survey was conducted from June through August 2015. Data on vaccination date for MCV1 and MCV2 and the child's date of birth were extracted from the child's vaccination certificate, which is a booklet containing information on all routine vaccinations received. The lists of target children for each sampled village were collected from the local township hospitals. Incidence of measles for each county was gathered from Guangxi CDC. Other information, such as population sizes and lists of counties, towns, and villages, were obtained from Guangxi Bureau of Statistics.

A database with suitable range checks and validation was developed in EpiData 3.1 (The EpiData Association, Odense, Denmark) to conduct double entry for the vaccination data. The integrity and validity of data were checked on each day of the survey.

### Outcome measurement

In China, the measles rubella (MR) vaccine and measles-mumps-rubella (MMR) vaccine are delivered to children as MCV1 and MCV2, respectively. Based on the difference between vaccination date and birth date, vaccination status for MCV1 and MCV2 was classified into timely, delayed, early, or unvaccinated. Timely vaccination for MCV1 requires that the child receives the vaccine at the age of 8 months (244–273 days).^26^ Timely vaccination for MCV2 is defined as the child receiving the vaccine between 18 and 24 months (548–730 days) after birth.^26^ If a child received a measles vaccine dose beyond the recommended schedule, it is defined as delayed vaccination. If the child received a measles vaccine dose before the recommended schedule, it is defined as early vaccination. Unvaccinated child means that the child was not vaccinated at the time of interview. Timely-and-complete measles vaccination means that the child receives both MCV1 and MCV2 on time.

According to the above definitions, coverage of early, timely, and delayed vaccination, unvaccinated coverage, and median delay period of vaccination were calculated separately for MCV1 and MCV2. In addition, timely-and-complete vaccination coverage was calculated.

### Statistical analysis

Sampling weights at each stage were calculated based on the probabilities of each sampling unit being selected in the sample.^38^, ^39^ Generally, sampling weight was the inverse of sampling proportion (i.e., the total amount of sampling unit at each level divided by the amount of the selected unit). Specifically, the sampling weight within the stratum was (the number of counties per stratum)/1, (the number of towns per county)/5 for the first stage of cluster sampling, (the number of villages per town)/6 for the second stage, and (the number of target children per village)/10 for the third stage. The final weight for each surveyed child was computed by multiplying the sampling weights at each sampling stage. Moreover, the finite population correction was applied to adjust for the variance of the vaccination coverage.^38^

Given the complex sampling design, the survey package in R 3.1.3 software was used to analyze the data. Vaccination coverage and 95% confidence intervals (CIs) were calculated. Weighted Kaplan–Meier analyses were used to determine the median delay vaccination period. Delayed vaccination was defined as the event of interest, and unvaccinated status was treated as censored data. The duration of delayed vaccination was the difference between the actual vaccination date and the upper limit of recommended date, and the duration of being unvaccinated was the difference between interview date and the upper limit of the recommended date. Children who received early or timely vaccination were excluded from this analysis, since they were no longer at risk of delayed vaccination.

Children aged less than 24 months were excluded from the calculation of median delay period of MCV2 and MCV2-related vaccination coverage.

The relationship between measles incidence and untimely coverage and delay period was explored graphically. Chi-squared test for trend was used to examine whether high-incidence regions had higher untimely vaccination coverage. Chi-squared test was also employed to compare the untimely vaccination coverage between regions.

### Ethical considerations

This study was approved by the Ethics Committees of Prince of Songkla University and Guangxi Medical University. Items of research purpose, research procedure, risk and benefit, confidentiality, right to refuse to participate or withdraw from the study at any time, on the informed consent form were explained fully to the child's primary guardian in each household. A consent form was signed by the primary guardian if he or she agreed voluntarily to participate in the study.

## Results

### Demographic characteristics of children

A total of 1216 eligible children were included in this study. Fifteen children did not have a vaccination certificate available at the time of interview (1.2%), and these children were substituted by other eligible children. Table 1 shows the demographic characteristics of the study sample. The ratio of boys to girls was approximately 55:45. Approximately 46.4% of the primary guardians were the child's grandparent or other relative. Nearly 92% of children were aged 25–48 months. Of the children, around 62.7% were Zhuang, 27.1% were Han, and 10.2% were other ethnicities.

**Table 1:** Demographic characteristics of children.

Characteristics	County				Total
	Longan	Zhaoping	Wuxuan	Longlin	
**Gender**					
Male	179 (57.9)	150 (50.5)	173 (56.2)	171 (56.6)	673 (55.3)
Female	130 (42.1)	147 (49.5)	135 (43.8)	131 (43.4)	543 (44.7)
**Ethnicity**					
Han	2 (0.6)	258 (86.9)	15 (4.9)	55 (18.2)	330 (27.1)
Zhuang	307 (99.4)	3 (1.0)	293 (95.1)	160 (53.0)	763 (62.7)
Other	0 (0.0)	36 (12.1)^a^	0 (0.0)	87 (28.8)^b^	123 (10.2)
**Child's primary guardian**					
Parent	180 (58.3)	164 (55.2)	172 (55.9)	136 (45.0)	652 (53.6)
Grandparent	127 (41.1)	133 (44.8)	135 (43.8)	163 (54.0)	558 (45.9)
Other relative	2 (0.6)	0 (0.0)	1 (0.3)	3 (1.0)	6 (0.5)
**Age, months**					
18–24	21 (6.8)	3 (1.0)	0 (0.0)	71 (23.5)	95 (7.8)
25–36	197 (63.8)	125 (42.1)	135 (43.8)	173 (57.3)	630 (51.8)
37–48	91 (29.4)	169 (56.9)	173 (56.2)	53 (17.5)	486 (40.0)
49–54	0 (0.0)	0 (0.0)	0 (0.0)	5 (1.7)	5 (0.4)
Total	309	297	308	302	1216

a Yao ethnicity.

b Miao ethnicity.

### Timeliness and completeness of vaccination without weighting

Table 2 presents the unweighted vaccination coverage. Regarding MCV1, the coverage was 4.6% (95% CI, 3.4%–5.8%) for early vaccination, 60.5% (95% CI, 57.8%–63.3%) for timely vaccination, and 33.7% (95% CI, 31.0%–36.3%) for delayed vaccination; 1.2% (95% CI, 0.6%–1.8%) were unvaccinated.

**Table 2:** Number and unweighted coverage of vaccination for MCV1 and MCV2 by county.

Vaccination status	Longan County		Zhaoping County		Wuxuan County		Longlin County		Overall	
	n (%)	95% CI	n (%)	95% CI	n (%)	95% CI	n (%)	95% CI	n (%)	95% CI
**MCV1**										
Early	15 (4.8)	2.5–7.3	14 (4.7)	2.3–7.1	7 (2.3)	0.6–3.9	20 (6.6)	3.8–9.4	56 (4.6)	3.4–5.8
Timely	224 (72.5)	67.5–77.5	191 (64.3)	58.9–69.8	146 (47.4)	41.8–52.9	175 (57.9)	52.4–63.5	736 (60.5)	57.8–63.3
Delayed	70 (22.7)	18.0–27.3	88 (29.6)	24.4–34.8	149 (48.4)	42.8–53.9	102 (33.8)	28.4–39.1	409 (33.7)	31.0–36.3
Unvaccinated	0 (0.0)	0.0–0.0	4 (1.4)	0.0–2.7	6 (1.9)	0.4–3.5	5 (1.7)	0.2–3.1	15 (1.2)	0.6–1.8
**MCV2**										
Early	11 (3.9)	1.6–6.0	10 (3.4)	1.3–5.5	9 (2.9)	1.0–4.8	18 (7.8)	4.3–11.2	48 (4.3)	3.1–5.5
Timely	265 (92.0)	88.9–95.1	248 (84.4)	80.2–88.5	236 (76.6)	71.9–81.3	163 (70.5)	64.7–76.4	912 (81.3)	79.1–83.6
Delayed	9 (3.1)	1.1–5.1	23 (7.8)	4.8–10.9	43 (14.0)	10.1–17.8	39 (16.9)	12.1–21.7	114 (10.2)	8.4–11.9
Unvaccinated	3 (1.0)	0.0–2.2	13 (4.4)	2.1–6.8	20 (6.5)	3.7–9.2	11 (4.8)	2.0–7.5	47 (4.2)	3.0–5.4
**MCV1 & MCV2**										
Timely-and-complete	192 (66.7)	61.2–72.1	162 (55.1)	49.4–60.8	116 (37.7)	32.3–43.1	105 (45.5)	39.0–51.9	575 (51.3)	48.4–54.2
Early, delayed or unvaccinated	96 (33.3)	27.9–38.8	132 (44.9)	39.2–50.6	192 (62.3)	56.9–67.7	126 (54.5)	48.1–60.9	546 (48.7)	45.8–51.6

CI, confidence interval; MCV1, first dose of measles-containing vaccine; MCV2, second dose of measles-containing vaccine.

**Fig. 2:**
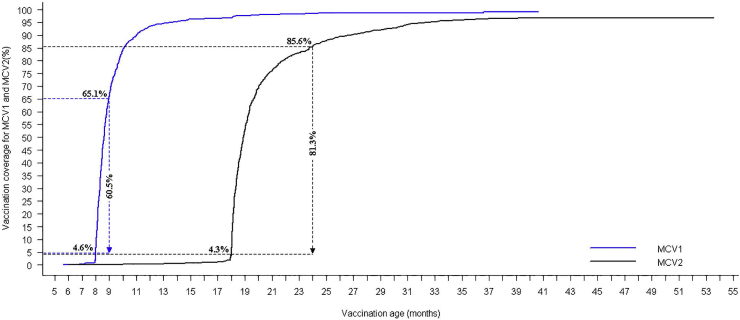
Cumulative vaccination coverage over time for MCV1 and MCV2. The dashed blue lines at the intercept of 4.6% and 65.1% are the cumulative coverage for MCV1 at 8 and 9 months, respectively. The vertical blue line corresponds to timely coverage (60.5%) for MCV1. The dashed black lines at the intercept of 4.3% and 85.6% are the cumulative coverage for MCV2 at 18 and 24 months, respectively. The vertical black line corresponds to timely coverage (81.3%) for MCV2. MCV1, first dose of measles-containing vaccine; MCV2, second dose of measles-containing vaccine. (For interpretation of the references to colour in this figure legend, the reader is referred to the web version of this article.)

For MCV2, approximately 81.3% (95% CI, 79.1%–83.6%) of the 1121 children aged 24 months or more at the time of interview received timely vaccination. In contrast, 4.3% (95% CI, 3.1%–5.5%) received early vaccination, 10.2% (95% CI, 8.4%–11.9%) received delayed vaccination, and 4.2% (95% CI, 3.0%–5.4%) were unvaccinated. Fig. 2 shows the unweighted cumulative vaccination coverage for MCV1 and MCV2 over the range of children's ages.

The overall timely-and-complete vaccination coverage was 51.3% (95% CI, 48.4%–54.2%), while 48.7% (95% CI, 45.8%–51.6%) of the children had early or delayed vaccination or were not yet vaccinated.

### Timeliness and completeness of vaccination with weighting

The weighted vaccination coverage is presented in Table 3. The coverage of MCV1 was 4.7% (95% CI, 3.9%–6.0%) for early vaccination, 58.4% (95% CI, 54.9%–62.0%) for timely vaccination, and 35.5% (95% CI, 32.4%–39.0%) for delayed vaccination; 1.4% (95% CI, 0.9%–2.0%) were unvaccinated. The rates of early, timely, delayed, and unvaccinated status for MCV2 were 4.7% (95% CI, 3.8%–6.0%), 76.9% (95% CI, 73.6%–80.0%), 13.9% (95% CI, 11.3%–17.0%), and 4.5% (95% CI, 3.4%–6.0%), respectively.

**Table 3:** Weighted vaccination coverage for MCV1 and MCV2 by county.

Vaccination status	Longan County (%)		Zhaoping County (%)		Wuxuan County (%)		Longlin County (%)		Overall (%)	
	Rate	95% CI	Rate	95% CI	Rate	95% CI	Rate	95% CI	Rate	95% CI
**MCV1**										
Early	5.8	3.9–8.0	4.3	3.1–6.0	2.1	1.4–3.0	6.2	4.7–8.0	4.7	3.9–6.0
Timely	73.7	69.3–78.0	65.5	56.9–73.0	49.8	44.1–55.0	56.1	50.7–61.0	58.4	54.9–62.0
Delayed	20.5	15.6–27.0	29.1	22.3–37.0	46.5	40.8–52.0	35.9	31.7–40.0	35.5	32.4–39.0
Unvaccinated	0.0	0.0–0.0	1.1	0.6–2.0	1.6	1.0–2.0	1.8	1.0–3.0	1.4	0.9–2.0
**MCV2**										
Early	3.8	2.5–6.0	4.1	2.8–6.0	2.1	1.3–3.0	7.7	5.8–10.0	4.7	3.8–6.0
Timely	93.1	90.3–95.0	85.1	81.0–88.0	76.4	72.7–80.0	66.4	60.0–72.0	76.9	73.6–80.0
Delayed	1.9	0.9–4.0	7.7	5.9–10.0	15.3	12.7–18.0	20.7	15.4–27.0	13.9	11.3–17.0
Unvaccinated	1.2	0.7–2.0	3.1	1.7–6.0	6.2	4.5–9.0	5.2	3.2–8.0	4.5	3.4–6.0
**MCV1 & MCV2**										
Timely-and-complete	68.4	64.0–73.0	56.3	46.3–66.0	39.7	36.0–43.0	40.6	35.3–46.0	47.4	44.0–51.0
Early, delayed or unvaccinated	31.6	27.5–36.0	43.7	34.3–54.0	60.3	56.6–64.0	59.4	53.8–65.0	52.6	49.2–56.0

CI, confidence interval; MCV1, first dose of measles-containing vaccine; MCV2, second dose of measles-containing vaccine.

The timely-and-complete vaccination coverage was 47.4% (95% CI, 44.0%–51.0%), while 52.6% (95% CI, 49.2%–56.0%) of children had early or delayed vaccination or were not yet vaccinated.

### Median delay vaccination period

**Fig. 3:**
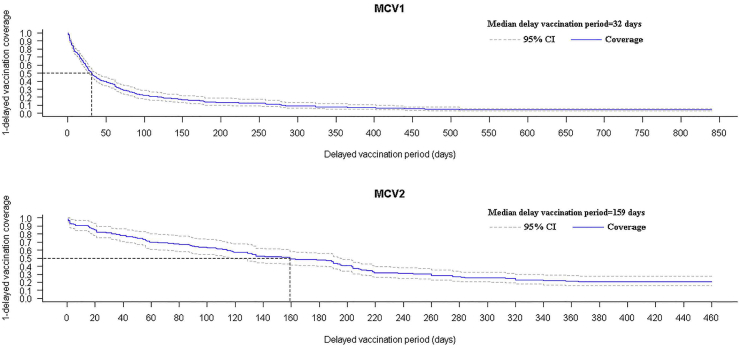
Weighted Kaplan–Meier curves of delayed vaccination for MCV1 and MCV2. The horizontal dashed-line is the median delayed vaccination coverage (50%). The vertical dashed-line is the median delay vaccination period (32 days for MCV1, 159 days for MCV2). MCV1, first dose of measles-containing vaccine; MCV2, second dose of measles-containing vaccine.

Table 4 shows the median delay period of measles vaccination. The overall median delay was 32 (95% CI, 27–38) days for MCV1 and 159 (95% CI, 118–195) days for MCV2. Fig. 3 shows the weighted Kaplan–Meier curves of delayed vaccination for MCV1 and MCV2.

**Table 4:** Median delay vaccination period for MCV1 and MCV2.

Surveyed county	MCV1		MCV2	
	Median delay period, days	95% CI	Median delay period, days	95% CI
Longan	20	14–29	189	128–247
Zhaoping	23	20–26	196	155–365
Wuxuan	35	31–40	204	153–236
Longlin	37	27–63	132	56–195
Overall	32	27–38	159	118–195

CI, confidence interval; MCV1, first dose of measles-containing vaccine; MCV2, second dose of measles-containing vaccine.

### Regional differences in untimely coverage, delay period, and measles incidence

**Fig. 4:**
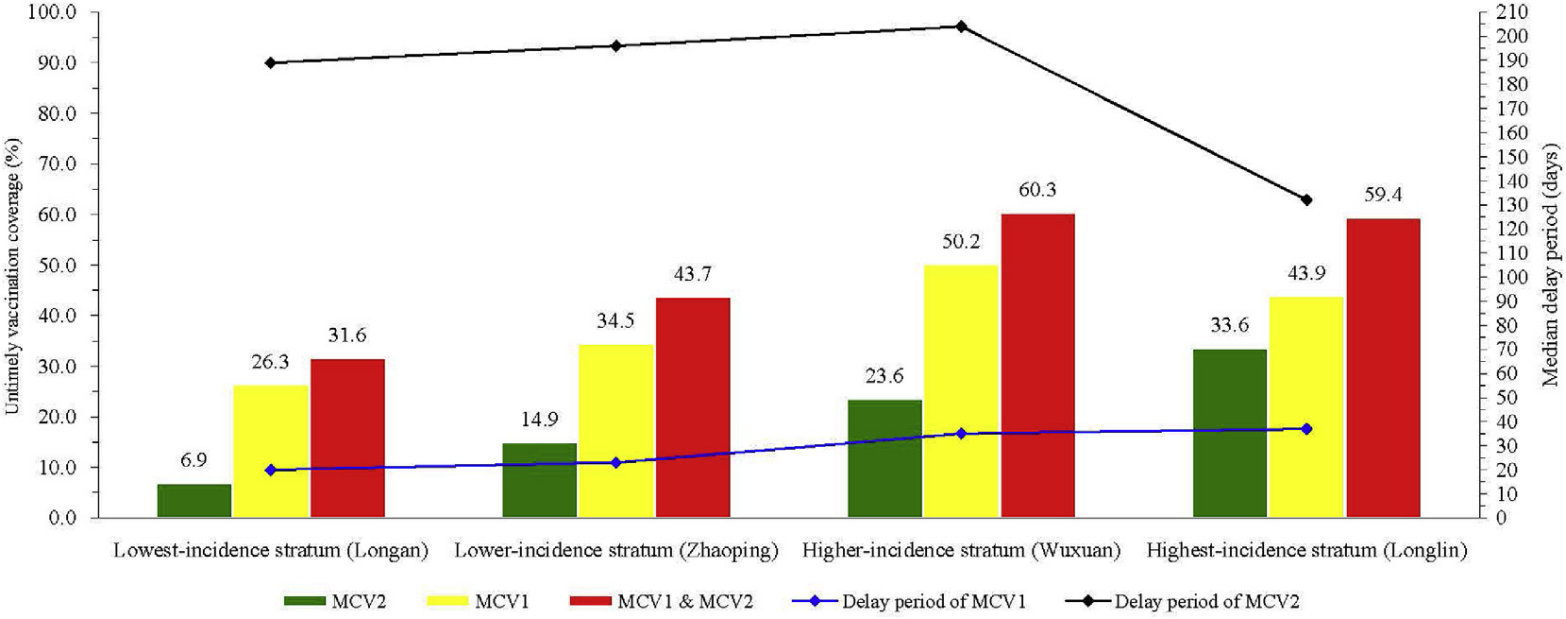
Regional differences in incidence, untimely coverage and delay period.

Fig. 4 summarizes the association between regional variation of measles incidence and heterogeneity of untimely coverage and delay period. There was a trend of high-incidence regions having higher untimely vaccination coverage (MCV1, MCV2, and MCV1 & MCV2; all *P* < 0.001 using Chi-squared test for trend), although there was no statistically significant difference between the higher-incidence and highest-incidence regions in MCV1 (*P* = 0.120) or MCV1&MCV2 (*P* = 0.800).

## Discussion

The findings of this study reveal that timely routine vaccination coverage of both MCV1 (58.4%) and MCV2 (76.9%) was low among children in rural Guangxi. The timely-and-complete coverage of measles vaccination was even lower (47.4%), and the median delay period of both MCV1 (32 days) and MCV2 (159 days) was long. Chinese health agencies often report simply the overall coverage in the official statistics and neglect the timeliness of vaccination, obscuring evidence of early and delayed vaccinations.^26^, ^34^ High vaccination coverage of measles is necessary but not sufficient for measles elimination. The timeliness and completeness of vaccination is an essential prerequisite for the progress towards global measles elimination and achievement of the Millennium Development Goal.^40^, ^41^ In a sense, the situation of untimely and incomplete vaccination is serious, and the timeliness and completeness of vaccination has become a major public health issue among children in rural Guangxi. Moreover, the findings of this study may shed some light on understanding the phenomenon of measles outbreaks in rural Guangxi, a region with high reported measles vaccination coverage.

Regarding the timeliness of MCV1, our findings are consistent with studies from other countries. In Argentina, around 55% of children aged 13–59 months received timely vaccination with MCV1, and 36% of children received delayed vaccinations.^27^ In Australia, the coverage of MCV1 among children aged 27–38 months was 58.1% for timely vaccination, 25.7% for delayed vaccination, and 10% for early vaccination.^28^ In Uganda, the coverage of MCV1 among children aged 10–23 months was 67.5% for timely vaccination, 21.8% for delayed vaccination, and 10.7% for early vaccination; 4.4% were unvaccinated.^31^ In Belgium, up to 32% of infants received MCV1 later than the recommended schedule.^32^ A study conducted among Swiss children found that approximately 62.6% of children aged 13 months were up to date for MCV1, and 59% of 25-month-old children received timely MCV2 vaccination.^35^

Unlike the study conducted by Bielicki in Switzerland,^35^ the timely vaccination coverage of MCV2 was higher than the timely coverage of MCV1 in our study. The possible reasons are multi-factorial. First, the timely vaccination period of MCV2 is recommended during 18–24 months of age, and the duration over which timely vaccination of MCV1 can be delivered is only 1 month.^26^ Thus, children have a wider period for vaccination with MCV2. Second, after delivering MCV1 to the children, the doctors at vaccination clinics usually make an appointment with the children's primary guardians on the personalized vaccination schedule of MCV2 to remind them of the dates to vaccinate.^34^, ^42^ Third, due to the trust in doctors, the primary guardians may be more concerned with the timely vaccination dates of MCV2 recommended by doctors.^43^

Our findings revealed the trend that the counties with higher measles incidence had poorer timeliness and completeness of vaccination and longer delays. In this light, differences in level of timeliness and completeness could be a possible explanation for the heterogeneity of measles incidence across study regions. Additionally, we found that counties with higher measles incidence also had higher proportions of children being cared for by non-parental guardians. With the development of the Sino-ASEAN economic zone, more and more parents are migrating from rural Guangxi to urban areas to find jobs. Such population migration resulted in large numbers of children left behind in rural hometowns. Previous study has highlighted that these children were more likely to suffer from health, education, and nutrition problems, due to the absence of parental care.^44^ Thus, the regional difference in proportions of parental migration may impact the heterogeneity of vaccination timeliness in this area, and these differences may possibly underlie the regional variation of incidence.

In terms of MCV2, although the highest-incidence region had the highest untimely vaccination coverage, the median period of delayed vaccination was the lowest in the highest-incidence region. This might be partly because supplementary immunization activities and catch-up immunization campaigns were frequently implemented in this region to reduce the period of delayed vaccination. However, the mechanisms underlying the disparity is complicated and will be explored in further study.

This was the first study to explore the timely-and-complete vaccination coverage of MCV among rural children. Furthermore, we studied not only the timely vaccination coverage, but also the median delay period of vaccination. Focusing on the timely coverage of MCV1, the timely coverage of MCV2, the timely-and-complete coverage of both MCV1 and MCV2, as well as the median delay period of vaccination, will be fundamental for further study of the vaccination coverage among children in rural China and will contribute to comprehensive insight into the officially-reported vaccination coverage. In addition, weighted estimates under the complex sampling design were calculated for the timely vaccination coverage and median delay period of vaccination.

Some limitations of this study should be mentioned. First, children without a vaccination certificate were excluded from the study. Those children might be more likely to be vaccinated early, late, or incompletely. Thus, the timeliness and completeness of measles vaccination may have been overestimated. Second, nearly half of the children in our study were the left-behind children, whose primary guardians were their grandparents or other close relatives, rather than parents. However, migrant children, who were born and grew up in rural areas but moved to urban or semi-urban areas with their parents, were not sampled in this study. Those migrant children might be likely pockets of delayed or incomplete vaccination. Third, determinants of failure to receive timely and complete measles vaccination among children in rural Guangxi were not revealed in this study. Vaccination of measles is one kind of health service, involving vaccination service providers, vaccination demanders, vaccination policies, and vaccination health systems. To better understand the effect of these factors on the timeliness and completeness of measles vaccination in rural Guangxi, a multi-level study would need to be conducted.

Despite these limitations, our findings have important implications for vaccination practice and policy making in China. The present study has revealed a necessity for improving the timeliness and completeness of measles vaccination among children in rural Guangxi. Measures to improve the coverage of timely-and-complete measles vaccination should be carried out urgently among the susceptible children in rural Guangxi. Moreover, the top priority should be given to early, delayed, unvaccinated, and incompletely vaccinated children. Additionally, incorporating the timeliness and completeness of vaccination into the official routine vaccination coverage statistics may assist in appraising the reported coverage of measles vaccination in China.

In summary, the timeliness and completeness of measles vaccination was low and the median delay period of measles vaccination was long among children in rural Guangxi. The discrepancy of high coverage of measles vaccination and large-scale outbreaks of measles strongly suggests that the timeliness and completeness of vaccination should be a serious concern in routine measles vaccination in rural China.

## Funding

This study was funded by the China Medical Board (14-202) and the National Natural Science Foundation of China (81502890). The sponsors had no role in study design, data collection, data analysis, or manuscript writing.

## Conflicts of interest

None declared.
